# Fraudulent Online Survey Respondents May Disproportionately Threaten Validity of Research in Small Target Populations

**DOI:** 10.1111/hex.14099

**Published:** 2024-06-06

**Authors:** Joshua H. Gordon, Kiyono Fujinaga‐Gordon, Christian Sherwin

**Affiliations:** ^1^ Department of Psychiatry, Perelman School of Medicine University of Pennsylvania Philadelphia Pennsylvania USA; ^2^ Department of Languages, Cultures and Applied Linguistics Carnegie Mellon University Pittsburgh Pennsylvania USA

To the Editor,

We would like to share our recent experiences with fraudulent study participants in response to a previous letter published in *Health Expectations* entitled ‘“Imposter participants” in online qualitative research, a new and increasing threat to data integrity?’ [1].

The widespread nature of the internet and the accessibility of online surveys have reduced barriers for collecting data from large samples of individuals. Nevertheless, it has become increasingly apparent that fraudulent activity is a critical threat to the validity of online data collection. We appreciate HEX's engagement in relatively small, bad‐faith respondents may have a proportionally greater impact on study validity. In the case of our study, we believe there was an extreme preponderance of fraudulent responses that exceeded many previous reports in the literature.

Between December 2023 and January 2024, we conducted a nationwide ethnographic questionnaire survey in the United States to characterize communities of Japanese speakers. We aimed to assess demographics, language attitudes, language use patterns, experiences of discrimination, health care access and mental health in this population. We used the ‘quota sampling’ method, ‘snowball technique’ [2] or ‘network sampling’ [3] by the PI reaching out to people through her professional networks and Japanese heritage community friends.

We opened the survey on 15 December 2023 using Qualtrics as a research survey tool. In the first 13 days of the study, we had 69 respondents. On 28 December 23, we approved an organization to reach out to their constituents via their social media accounts (Facebook and Instagram). That day, we had 1774 survey responses. After our research team reviewed the results, our suspicions were quickly raised about the possibility of fraud based on observations of repetitive answers, answers not consistent with questions being asked and numerous emails sent to study PI asking about survey compensation, as well as many completely blank emails, consistent with concerning features reported by authors in HEX. We contacted our IRB about our concerns and closed the survey at 10 pm on the night of 28 December. We reopened our survey after obtaining IRB approval to adjust the language of our survey, adding language stating that we would be evaluating responses for authenticity.

During our data analysis, we utilized the following criteria to classify responses based on suspected fraud or authenticity: (1) responses that appeared to be duplicate submissions from the same individual (i.e., multiple submissions with identical answers or with the same name listed); (2) low effort, repetitive submissions (e.g., answering 1 for all Likert scale questions or ‘n/a’ for open‐ended responses); (3) nonsensical submissions; (4) submissions where survey responses were inconsistent with eligibility criteria (i.e., not listing Japanese as a spoken language, since the survey was aimed at surveying Japanese speakers); (5) responses not internally consistent (e.g., mismatch between reported age and birth year).

Based on these criteria, we classified 356 responses as authentic and 1487 as suspected. During the first 12 days of our survey being open, 12/69 (17/4%) were classified as suspected fraud. After being posted to social media, we classified 1475/1774 (83.1%) as suspected fraud. Nevertheless, there is a significant likelihood that our criteria were not able to correctly classify all fraudulent responses. Considering fraud classification in terms of test sensitivity and specificity, it is apparent that the higher the proportion of fraudulent responses, the more likely that fraudulent responses would be misclassified as authentic, and that these misclassified responses would have a greater impact on study results. To illustrate this point, if we consider a hypothetical classification strategy that has a 90% sensitivity and specificity to detect fraudulent responses, assuming a prevalence of 50% fraud, approximately 90% of responses we determine to be authentic will truly be authentic. However, if the prevalence of fraud is 80%, only approximately 69% of responses classified as authentic would be truly authentic; at 90% fraud, the proportion of truly authentic responses decreases to 50%. As such, we conjecture that the potential impact of fraud is a yet greater threat to validity in studies in which the population of interest is small.

In addition to the threats to study validity, we wish to acknowledge the impact of this experience on investigators. It is disheartening to encounter issues related to fraud during research. Our team experienced significant demoralization related to this occurrence. Although we would hope that this is not something any researcher would encounter, we are concerned that this may be an issue that is here to stay. Moreover, as technology in AI‐based language processing continues to improve and wider access, the sophistication of fraudulent respondents may become even more difficult to identify. Successful strategies have been described elsewhere such as providing regional‐based compensation, requiring a physical mailing address, and conducting interviews over phone [1, 4]. Additional strategies are needed to continue to address these issues and minimize the impact of fraudulent responses on future studies.

## Author Contributions

**Joshua H. Gordon:** conceptualization, formal analysis, investigation, writing–original draft, writing–review and editing, methodology. **Kiyono Fujinaga‐Gordon:** conceptualization, data curation, investigation, methodology, project administration, writing–review and editing, funding acquisition. **Christian Sherwin:** formal analysis, investigation, writing–review and editing.

## Conflicts of Interest

The authors declare no conflicts of interest.

## Data Availability

Research data are not shared.
